# Serum Interleukin-6 as an Early Indicator of Trauma Complications

**DOI:** 10.7759/cureus.68606

**Published:** 2024-09-04

**Authors:** Aparna Laishram, Alice Ruram, Bhaskar Borgohain, Kanchana Laishram

**Affiliations:** 1 Department of Biochemistry, North Eastern Indira Gandhi Regional Institute of Health and Medical Sciences, Shillong, IND; 2 Department of Orthopedics, North Eastern Indira Gandhi Regional Institute of Health and Medical Sciences, Shillong, IND

**Keywords:** interleukin (il)-6, injury severity score, trauma, complications, sensitivity and specificity

## Abstract

Background: Trauma is a major global health issue, associated with high mortality and complications like inapparent hypoxia, fat embolism syndrome (FES), sepsis, and multiple organ dysfunction syndrome (MODS). Early identification of high-risk patients is crucial but challenging. Serum interleukin-6 (IL-6), a key inflammatory cytokine, has shown potential as a biomarker for predicting adverse outcomes in trauma. IL-6 levels typically increase rapidly following trauma, peaking within 12 to 24 hours. Despite its potential role, there is limited research on the effectiveness of IL-6 as an early marker for trauma-related complications. This study aims to assess whether monitoring serum IL-6 levels at specific intervals after trauma can aid in early risk assessment and predict the development of these complications.

Materials and methods: This prospective observational cohort study at North Eastern Indira Gandhi Regional Institute of Health and Medical Sciences (NEIGRIHMS) included 119 trauma patients aged 19-65 years, admitted within 12 hours of injury. Venous blood samples (5 mL each) were collected at 12 and 24 hours for IL-6 and C-reactive protein (CRP) analysis. Injury severity score (ISS) was assessed for all the patients upon arrival to the emergency department at NEIGRIHMS and was categorized as mild, moderate, severe, and very severe. Inapparent hypoxia, FES, sepsis, and MODS were assessed using pulse oximetry, Gurd’s criteria, quick sequential organ failure assessment (qSOFA) score, and Marshall's multiple organ dysfunction score, respectively.

Results: Among the participants, 21.85% developed complications; primarily inapparent hypoxia. Serum IL-6 levels were significantly elevated in individuals with complications at both 12 hours (p < 0.001) and 24 hours (p < 0.001) post-trauma. At the 12-hour mark, serum IL-6 demonstrated a sensitivity of 92.3% and a specificity of 78.5%, with a cut-off value of 37.26 pg/mL. By 24 hours, the sensitivity increased to 96.2% and the specificity to 87.1%, with a cut-off value of 55.08 pg/mL. Patients with MODS had the highest IL-6 levels, with medians of 270.87 pg/mL at 12 hours and 826.10 pg/mL at 24 hours. A strong correlation was observed between serum IL-6 at 24 hours and the ISS (r_s_ = 0.725, p < 0.001). At 12 hours, there was a moderate correlation between serum IL-6 and CRP (r_s_ = 0.488, p < 0.001). By 24 hours, this correlation strengthened to a strong level (r_s_ = 0.749, p < 0.001).

Conclusions: The significant association of serum IL-6 levels with both ISS and CRP highlights its potential role in assessing trauma severity. The high sensitivity and specificity of IL-6 at the 24-hour make it a valuable biomarker for the early detection of trauma-related complications.

## Introduction

Trauma and its associated complications present significant global health challenges, characterized by high mortality rates. In India, road traffic accidents saw an 11.9% increase in 2022, with fatalities rising by 9.4% and injuries by 15.3% compared to the previous year [1]. Effective early assessment and monitoring upon hospital arrival are crucial for predicting 30-day survival outcomes [2]. Trauma patients often face severe complications, including apparent hypoxia, fat embolism syndrome (FES), sepsis, and multiple organ dysfunction syndrome (MODS), which can be life-threatening and difficult to manage [3].

MODS and sepsis are major contributors to adverse outcomes in trauma patients, exacerbating the healthcare burden through high treatment costs [4,5]. Early intervention has been shown to reduce both mortality and healthcare expenses [6]. FES, frequently occurring in patients with long bone fractures, remains under-recognized and lacks a definitive diagnostic test. Hypoxia, commonly observed in trauma patients, may indicate early FES, though further investigation is needed to clarify this association [7]. The challenge of early and accurate risk assessment persists due to the complex and often delayed presentation of trauma-related complications.

Biomarkers play a crucial role in predicting trauma complications. Among various biomarkers explored for their potential to predict trauma-related complications, interleukin-6 (IL-6) has emerged as a promising candidate. IL-6 is one of the main inflammatory cytokines that is released by the body in response to tissue injury and inflammation. This multifunctional cytokine was initially identified as B-cell stimulatory factor 2 (BSF-2) and later renamed IL-6 in 1988 [8]. It is produced by various cell types, including macrophages, endothelial cells, and fibroblasts, playing a pivotal role in the regulation of inflammation. It belongs to the four-α helical bundle cytokine family and interacts with IL-6Rα and gp130 to form a hexameric complex [9]. IL-6 signaling is mediated through JAK1 and STAT3 phosphorylation, sharing similar pathways with other cytokines in its family [8,10].

Elevated IL-6 levels have been linked to severe outcomes in trauma and critically ill patients [11,12]. Patients with minor injuries exhibit lower IL-6 levels compared to those with severe injuries. IL-6 is found to progressively increase over the first six hours after trauma in severe and very severe injuries with significant differences in the peak IL-6 level among different injury severity groups. This trend continues up to 12 hours post-injury, while the levels may decrease by 48 hours [13,14]. As an early marker for complications that may develop after trauma, IL-6 may provide a more timely predictive tool compared to C-reactive protein (CRP), which has a slower response time, although it is also indicative of inflammation [11,15,16].

So far, very few studies have been conducted to assess the potential of serum IL-6 as an early marker of trauma complications. IL-6 levels are known to rise rapidly in response to trauma and typically peak within 12 to 24 hours following trauma. Research has indicated that measuring serum IL-6 concentrations within the first 24 hours after trauma may be valuable for predicting post-traumatic complications, especially multiple organ failure. It has also been found that IL-6 is associated with tissue hypoxia and cellular stress response and can precede clinical signs of sepsis, making it an important tool for the diagnosis of sepsis [3].

Given the limited research and conflicting findings regarding the use of IL-6 as an early marker for trauma complications and recognizing the critical need for early detection and management of such complications, this study aims to assess the effectiveness of serum IL-6 levels in predicting adverse outcomes following trauma.

## Materials and methods

This study is a hospital-based, prospective observational cohort investigation conducted for a period of 20 months from November 2022 to June 2024. A total of 119 patients aged 19 to 65 years, admitted at North Eastern Indira Gandhi Regional Institute of Health and Medical Sciences (NEIGRIHMS) hospital within 12 hours of experiencing trauma with skeletal injury were included by employing the method of convenience sampling. Patients with a history of uncontrolled cardiac diseases, respiratory conditions, chronic systemic illnesses, diabetes mellitus, rheumatoid arthritis and patients currently undergoing steroid therapy were excluded as they are possible confounding factors. Based on the correlation coefficient of 0.61 for IL-6 with ISS in the prediction of complications following trauma, with a power of 80% and an alpha error of 5%, the sample size was estimated to be 116, using nMaster 2.0 (Informer Technologies, Inc., USA).

After obtaining informed consent, participants underwent a comprehensive assessment, including history, vital signs, clinical examination, and laboratory investigations. At 12 and 24 hours post-injury, blood samples were collected from the study participants for the measurement of serum IL-6 and CRP. Serum IL-6 levels and serum CRP levels were assessed using the Roche Cobas e601 electrochemiluminescence system (Roche Diagnostics, Switzerland) and Beckman Coulter Immage 800 nephelometer system (Beckman Coulter, Inc., USA), respectively. Quality control was maintained using Bio-Rad internal quality control samples and external quality control samples provided by ACBI CMC Vellore. Ethical clearance was obtained from the Institution Ethics Committee, NEIGRIHMS on October 22, 2022, with Ref No. NEIGR/IEC/M7/T1/2022 (Thesis No: T98/2022/98).

Injury severity score (ISS) was assessed for all the patients upon arrival to the emergency department at NEIGRIHMS and the patients were categorized based on the scores, with a score of less than 9 as mild, 9 to 15 as moderate, 16 to 25 as severe, and more than 25 as very severe [17]. The assessment of inapparent hypoxia, FES, sepsis, and MODS was done based on pulse oximetry (SpO2 ≤ 94%), Gurd’s criteria [7], quick sequential organ failure assessment (qSOFA) score [18] and Marshall’s multiple organ dysfunction score [19], respectively.

The demographic details of the study participants have been expressed in frequency and percentage and the levels of biomarkers in the study participants are given as median values along with interquartile ranges. Mann-Whitney U test was used to compare the means and tested significance between patients with and without complications. Receiver operating characteristic (ROC) curve analysis was done to assess the sensitivity and specificity of serum IL-6 and CRP at 12 hours and 24 hours post-injury in predicting the development of trauma complications and the cut-off values were determined by analysis of the coordinates of the curves using Youden’s index. The association between serum IL-6, serum CRP, and ISS was determined using Spearman’s correlation analysis. IBM SPSS version 29.0 (IBM Corp., Armonk, NY) was used for the statistical analysis, and a p-value of < 0.05 was taken to be statistically significant.

## Results

Out of the total 119 patients, 26 patients, constituting 21.85%, experienced complications, while the remaining majority, comprising 93 patients (78.15%), did not encounter any complications. The largest proportion of participants falls within the age group of 19 to 25 years (33.61%). The majority of participants were males (85.71%), with females comprising only 14.29%. Most of the trauma cases were caused by road traffic accidents (86.55%) and the majority of them (53.78%) had moderate ISS (Table 1).

**Table 1:** Demographic details of the study participants

Demographic parameters	Patients who did not develop any complications (N = 93)	Patients who developed complications (N = 26)	All patients (N = 119)	Percentage (N=119) (%)
Age (years)				
19-25	35	5	40	33.61
26-35	29	7	36	30.25
36-45	8	4	12	10.08
46-55	15	6	21	17.65
56-65	6	4	10	8.40
Male	80	22	102	85.71
Female	13	4	17	14.29
Cause of trauma				
Road traffic accident	79	24	103	86.55
Fall from height	14	2	16	13.45
Injury severity score				
Mild	38	0	38	31.93
Moderate	53	11	64	53.78
Severe	2	7	9	7.56
Very severe	0	8	8	6.72

Patients with MODS had the highest serum IL-6 levels, with medians of 270.87 (48.28-496.95) pg/mL at 12 hours and 826.10 (254.67-1,120.02) pg/mL at 24 hours, and the highest serum CRP levels, with medians of 1.24 (0.55-5.19) mg/dL at 12 hours and 8.97 (7.04-37.57) mg/dL at 24 hours. Only one patient developed subclinical FES and the median values for FES were not assessed. All patients who developed complications also exhibited inapparent hypoxia, except for two cases of sepsis (Table 2).

**Table 2:** Comparison of median serum IL-6 and CRP levels at 12 and 24 hours post-injury with interquartile ranges across various complications FES - fat embolism syndrome, sepsis, MODS - multiple organ dysfunction syndrome, IL-6 - interleukin-6, CRP - C-reactive protein

Complication	No. of patients	IL-6 at 12 hours (pg/mL)	IL-6 at 24 hours (pg/mL)	CRP at 12 hours (mg/dL)	CRP at 24 hours (mg/dL)
Inapparent hypoxia	24	61.84 (39.41-158.25)	143.85 (86.43-271.60)	0.81 (0.49-2.13)	8.04 (5.90-10.61)
Sepsis	12	125.72 (43.05-421.77)	239.50 (103.67-785.17)	1.23 (0.469-3.88)	8.18 (6.48-17.89)
MODS	4	270.87 (48.28-496.95)	826.10 (254.67-1120.02)	1.24 (0.55-5.19)	8.97 (7.04-37.57)
FES	1	50.74	81.07	1.28	6.24
Death	0	--	--	--	--

**Figure 1:**
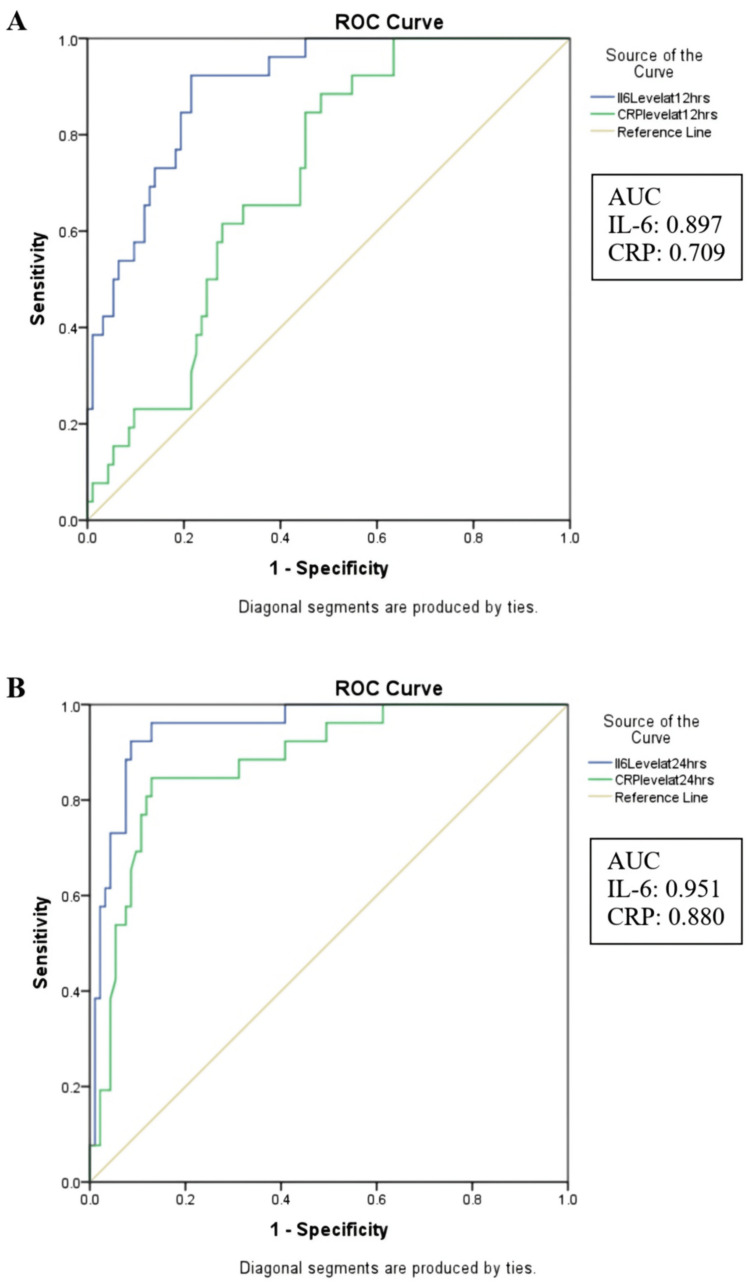
Receiver operating characteristic (ROC) curve analysis for predicting trauma complications using serum IL-6 and serum CRP (A) ROC curves of serum IL-6 and CRP at 12 hours post-trauma, (B) ROC curves of serum IL-6 and CRP at 24 hours post-trauma. IL-6 - interleukin-6, CRP - C-reactive protein

The mean serum IL-6 levels were significantly higher in patients who developed complications compared to those without complications, both at 12 hours (p < 0.001) and 24 hours (p < 0.001). The ROC curves illustrate that the area under the curve (AUC) for serum IL-6 at 12 hours is 0.897, which exceeds the AUC of 0.709 for CRP at the same time point. Furthermore, at 24 hours, the AUC for serum IL-6 rises to 0.951, exceeding the AUC of 0.880 for CRP at this time point (Figures 1A, 1B). The coordinates of the curves were analyzed and using Youden’s index, at a cut-off value of 37.26 pg/mL, the sensitivity and specificity of serum IL-6 at 12 hours are found to be 92.30% and 78.50%, respectively. At 24 hours, serum IL-6, with a cut-off value of 55.08 pg/mL, demonstrates a sensitivity of 96.2% and a specificity of 87.1% (Table 3).

**Table 3:** Predictive values of serum IL-6 at 12 and 24 hours for trauma-related complications PPV - positive predictive value, NPV - negative predictive value, AUC - area under the curve, IL-6 - interleukin-6

Test parameters	Cut-off value	Sensitivity (%)	Specificity (%)	PPV (%)	NPV (%)	AUC
IL-6 (pg/mL) (12 hours)	37.26	92.3	78.5	54.5	97.3	0.897
IL-6 (pg/mL) (24 hours)	55.08	96.2	87.1	67.6	98.8	0.951

**Figure 2:**
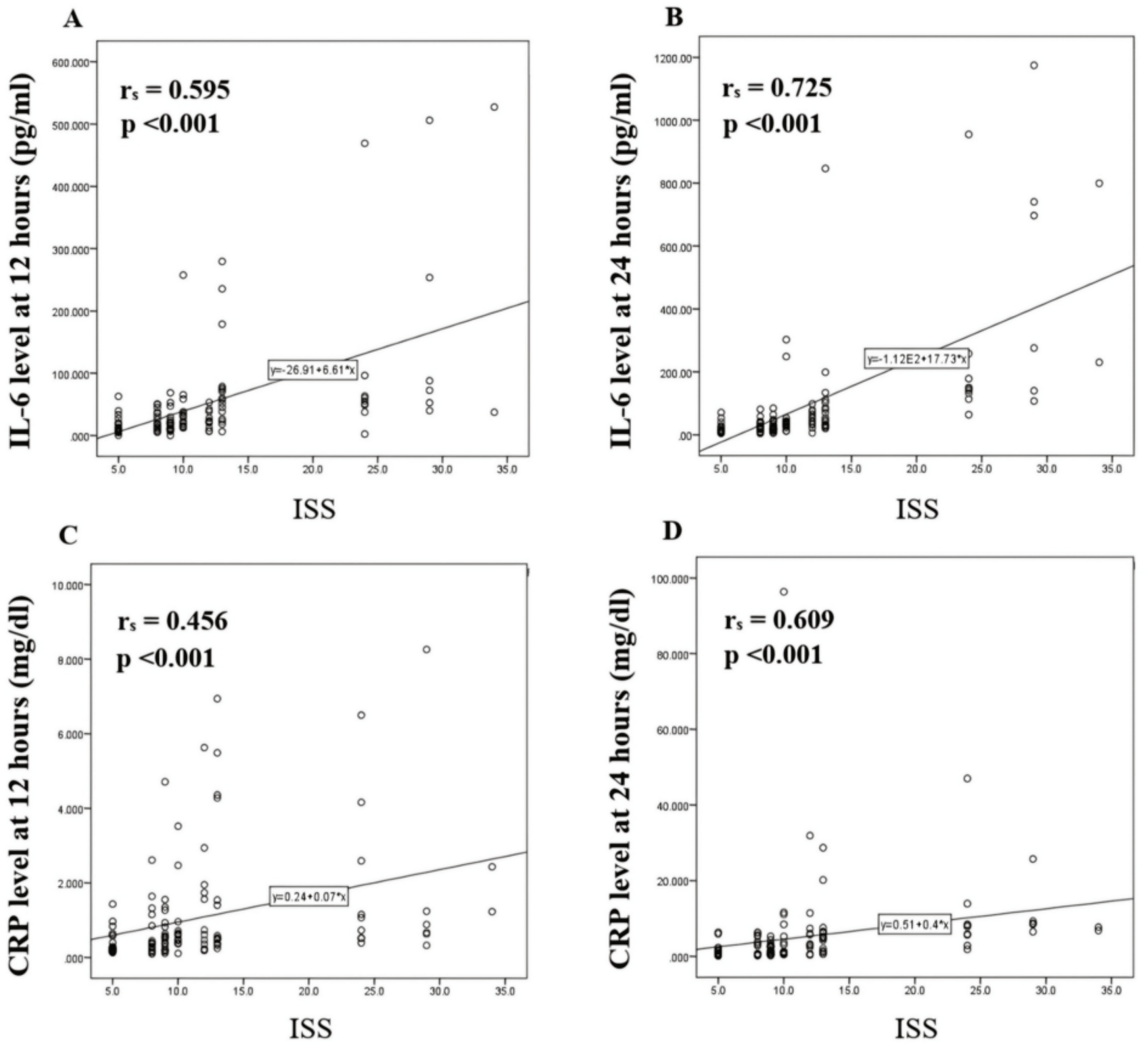
Scatter plots of serum IL-6 levels (pg/mL) and serum CRP levels (mg/dL) versus Injury Severity Score (ISS) in study participants (A) Correlation between serum IL-6 and ISS at 12 hours, (B) between serum IL-6 and ISS at 24 hours, (C) between serum CRP and ISS at 12 hours, and (D) between serum CRP and ISS at 24 hours post-trauma. IL-6 - interleukin-6, CRP - C-reactive protein

A moderate positive correlation was observed between serum IL-6 and ISS at 12 hours post-injury (r_s_ = 0.595, p < 0.001) as well as between serum CRP and ISS at both 12 hours (r_s_ = 0.456, p < 0.001), and 24 hours post injury (r_s_ = 0.609, p < 0.001). However, a strong positive correlation is seen between serum IL-6 and ISS at 24 hours post-injury (r_s_ = 0.725, p < 0.001) (Figures 2A-2D).

We also investigated the association between serum IL-6 and serum CRP, and we found a moderate positive correlation between these biomarkers at 12 hours (r_s_ = 0.488, p < 0.001), which strengthens to a strong correlation at 24 hours post-injury (r_s_ = 0.749, p < 0.001) (Table 4).

**Table 4:** Correlation between serum IL-6 and serum CRP in the study participants *Spearman rank correlation IL-6 - interleukin-6, CRP - C-reactive protein

Variable	Correlation Coefficient (r_s_)*	P-value
IL-6 and CRP at 12 hours	0.488	<0.001
IL-6 and CRP at 24 hours	0.749	<0.001

## Discussion

Most participants in our study were males, aged 19 to 25 years, with road traffic accidents as the leading cause, indicating a higher risk of traumatic injuries in this younger demographic. Road accidents among the young population should be properly addressed to reduce the associated morbidity and mortality. Out of the 26 patients who developed complications, inapparent hypoxia was the most common, and similar results were noted by Laishram et al. [20]. The importance of continuous pulse oximeter monitoring in patients with long bone fractures to detect early pulmonary complications was also emphasized by Wong et al. [7].

Serum IL-6 and CRP levels were highest in patients with MODS at both 12 hours and 24 hours after injury. Our findings align with the documented progressions from sepsis to systemic inflammatory response syndrome and eventually to MODS, highlighting the role of these biomarkers in the mechanisms behind systemic inflammation and organ failure. Serum IL-6 levels were found to be significantly higher in patients with complications compared to those who did not develop any complication at both time points (p < 0.001), while CRP showed a slightly lower significance at 12 hours (p = 0.001) compared to 24 hours (p < 0.001). A similar finding was observed by Volpin et al. in their study, with significantly elevated IL-6 and IL-8 levels in injured patients compared to healthy controls, while differences in other cytokines were not significant [21]. Qiao et al. also found that IL-6 levels within 24 hours post-trauma were notably higher among patients who experienced complications or mortality [3]. Similarly, in one of the studies by Tocu et al., levels of biomarkers PCT and IL-6 were significantly higher in patients who did not survive compared to those who did, whereas the difference was not significant for CRP [22]. This evidence indicates the potential of IL-6 over CRP for predicting trauma complications.

Upon assessing the association between serum IL-6 and serum CRP with ISS in the study participants, a significant increase in IL-6 and CRP levels at both 12 hours and 24 hours with higher ISS was observed in the participants. At 24 hours post-injury, a strong positive correlation was found between serum IL-6 and ISS. This highlights the role of serum IL-6 and CRP in the mechanism of generation of systemic inflammation by the body’s immune system, with IL-6 being one of the main drivers of the inflammatory process. Elevated IL-6 levels upon arrival at the hospital were found to be associated with longer ICU stays and higher abbreviated injury scores by Taniguchi et al., suggesting the utility of IL-6 in early trauma assessment [23]. Other studies also noted that increased levels of IL-6 significantly correlated with the severity of trauma and patient outcomes [13,24].

IL-6 was found to have higher sensitivity and specificity in predicting the risk of development of complications compared to CRP at 12 hours and 24 hours post-injury. At 24 hours, with a cut-off value of 55.08 pg/mL, serum IL-6 showed a sensitivity of 96.2% and a specificity of 87.1%. Picod et al. demonstrated that IL-6 has prognostic superiority when compared to CRP and they came to the conclusion that when both biomarkers are analyzed together, incorporating CRP adds minimal benefit compared to IL-6 alone [25]. Similarly, Laishram et al. found that elevated IL-6 levels within 24 hours post-surgery predicted post-operative complications with a sensitivity of 75% [20]. However, in slight contrast to our results, Prakash et al. observed that elevated serum IL-6 levels at 12 hours post-trauma are associated with an increased likelihood of developing FES, and no significant correlation was found between IL-6 levels at six or 24 hours and FES development. At 12 hours post-injury, they noted that IL-6 had a sensitivity of 73% and specificity of 92% for diagnosing FES at a cut-off value of 134 pg/mL [26]. 

In our study, serum IL-6 and serum CRP exhibit a significant moderate positive correlation with each other at 12 hours post-injury, while at 24 hours, there is a strong positive correlation between these two biomarkers. This indicates that as time progresses from the initial trauma, the association between serum IL-6 and CRP levels increases, suggesting a synchronized inflammatory response. Previous studies have suggested that the secretion of CRP by the liver is primarily stimulated by IL-6, and it is regulated at the transcriptional level by IL-6 [15,16]. Further exploration in this regard may yield results that can help better understand the underlying mechanism.

Overall, early measurement of serum levels of IL-6 and CRP post-trauma is shown to be effective in predicting trauma complications. Our results show that serum IL-6 level at 24 hours is a better marker compared to the levels at 12 hours with higher sensitivity and specificity. The role of IL-6 in detecting patients at risk of complications should be further explored as it is a potential biomarker in the early assessment of trauma patients.

The study on serum IL-6 as a biomarker for trauma complications has notable limitations. It involved a small cohort of 119 predominantly young male trauma patients, which may limit the generalizability to other demographics. IL-6 levels were assessed only at 12 and 24 hours post-injury, potentially missing important long-term data. The observational nature of the study prevents establishing causality. Due to the small sample size for each trauma-related complication, multivariate analysis could not be conducted. This analysis would have controlled for confounding factors, potentially enhancing predictive accuracy and enabling better risk stratification. Addressing these limitations in future research could improve the understanding of IL-6's role in trauma care.

## Conclusions

The high sensitivity and specificity of IL-6 at 24 hours post-trauma make it a valuable biomarker for the early detection of trauma-related complications like inapparent hypoxia, FES, sepsis, and MODS. IL-6 may be more effective than CRP or ISS alone in identifying patients at risk of developing these complications. While both IL-6 and CRP are valuable for assessing injury severity, IL-6 shows a stronger correlation as time progresses. Integrating serum IL-6 measurements into trauma management protocols could improve patient care by enabling more timely and targeted interventions. However, additional research is needed to fully validate the role of IL-6 in evaluating trauma patients.
